# Development and validation of the short form of the Parker personality measure among Chinese college students

**DOI:** 10.3389/fpubh.2025.1685858

**Published:** 2025-11-13

**Authors:** Yihua Zhang, Jing Huang, Yangjie Chen, Jiale Xu, Runtang Meng, Bingren Zhang

**Affiliations:** 1 Affiliated Hospital (School of Clinical Medicine), Hangzhou Normal University, Hangzhou, China; 2 School of Public Health and Nursing, Hangzhou Normal University, Hangzhou, China

**Keywords:** Parker personality measure, college students, pathological personality, psychometric properties, cross-cultural validation

## Abstract

**Objective:**

Current diagnostic frameworks for pathological personality are shifting from categorical to dimensional models. While the Personality Inventory for DSM-5 represents the most extensively validated dimensional measure across Western and Eastern cultures, its length limits practical utility. This study developed the Parker Personality Measure Short Form (PERM-SF) to facilitate efficient screening of pathological personality traits.

**Methods:**

Two samples of college students (Sample 1: *N* = 1,768; 937 women and 831 men, aged 20.59 years ± 1.95, ranging from 16 to 27 years; Sample 2: *N* = 1,614; 887 women and 727 men, aged 20.38 years ± 1.59, 17–27 years) were recruited consecutively from one university in China through online random sampling. Exploratory factor analysis in Sample 1 identified items with robust psychometric properties for inclusion in the PERM-SF. Confirmatory factor analysis in Sample 2 validated the factor structure. Subsequent analyses evaluated internal consistency, longitudinal measurement invariance, and construct validity.

**Results:**

Thirty-eight of the 92 items of PERM were deleted due to low factor loadings or high cross loadings, resulting in a five-factor model consisting of 54 items (F1: Dissociality, F2: Self-doubt-Detachment, F3: Disinhibition-Negative affectivity, F4: Anankastia, F5: Borderline pattern). All factors demonstrated good internal consistency (McDonald’s ω = 0.834–0.932). Longitudinal measurement invariance held at strict level (ΔCFI ≤ 0.001, ΔRMSEA ≤ 0.001) and two-way random-effects ICCs ranged from 0.488 to 0.759. Validity analyses revealed significant weak-to-moderate correlations with the Symptom Checklist-90 and established discriminant validity for all factor pairs, as supported by the Heterotrait-Monotrait Ratio test.

**Conclusion:**

The PERM-SF exhibits adequate preliminary psychometric properties for rapid assessment of pathological personality dimensions, supporting its utility for research and screening applications at least among Chinese young adults.

## 1 Introduction

Personality is a composite reflecting an individual’s stable cognitive, emotional-affective, and behavioral patterns, encompassing past influences, interpretations of the present, and constructs for the future (1). The spectrum from a healthy personality to severe personality disorders forms a continuum that reflects varying degrees of pathological personality (2). A recent global systematic review and meta-analysis estimated the worldwide pooled prevalence of personality disorders (PDs) at 7.8% (3). Despite marked regional variations with reported prevalence rates of 4.1% in Asian populations (4), significantly lower than the 12.16% observed in Western countries (5), PDs contribute substantially to functional impairment, reduced productivity, and disease burden (6). Furthermore, PDs exhibit high comorbidity with other psychiatric conditions, exacerbating risks of premature mortality and suicidal behavior (2). Primary and secondary prevention strategies on personality disorders can monitor of risk factors operating at the population level, e.g., use of scales, and are needed to reduce the individual and societal burdens in the community (7). Therefore, to optimize patient outcomes and recovery, it is essential to assess pathological personality and identify personality disorders at an early stage.

Personality disorder (PD) diagnostic systems fall into two main categories. The first is the categorical diagnostic system of PD based on typical experiences and behaviors, with representative assessment tools such as the Personality Diagnostic Questionnaire-5 (8) and the Structured Clinical Interview for Diagnostic and Statistical Manual of Mental Disorders, Fifth Edition (DSM-5) Personality Disorders (9), based on the widely used DSM-5. However, this categorical system faces many practical problems [e.g., (6, 10)]. Firstly, the number of independent PD types is uncertain, and there are no clear boundaries between types and severity of PD. Additionally, the clinical presentation of patients with specific types of PD varies across cultures. For example, patients with borderline personality disorder under religious cultures had fewer addictive behaviors (11). Similarly, while obsessive-compulsive personality disorder in Western contexts is characterized by order, perfectionism, and control stemming from internal conflicts, its manifestation in Chinese patients is often associated with culturally shaped traits such as dependency, conformity, and self-restraint (12). Furthermore, different types of PD diagnosis present various issues of clinical utility (13). For instance, although effective evidence-based therapies are available for borderline personality disorder, there is a stigma attached to this group by mental health professionals (14), which might delay effective treatment and pose a lethal risk (15). Conversely, for other personality disorder types like antisocial, narcissistic, and avoidant, evidence of effective psychotherapy is limited (16).

Therefore, an alternative model has been adopted in both the DSM-5 Section III [the alternative model, DSM-5-AMPD, (9)] and the latest International Classification of Diseases-11 [ICD-11, (17)], namely the PD dimensional diagnostic system. This system places greater emphasis on assessing a patient’s core personality traits and the degree of impaired functioning rather than meeting specific PD symptoms (18, 19). Current instruments for assessing pathological personality traits mainly include the Personality Inventory for DSM-5 [PID-5, (18)], serial questionnaires based on the ICD-11 by Oltmanns and Widiger (20–22), and the Personality Assessment Questionnaire for ICD-11 (23), etc. The PID-5 is possibly the best available dimensional trait assessment tool, validated across diverse Western and Eastern cultures (24), demonstrating reliability and validity in capturing pathological personality traits per both DSM-5-AMPD and ICD-11 criteria (25), and it has both self-report (18) and informant-report (26) versions. However, the PID-5 comprises 220 items. During self-report, patients with PD often exhibit emotional instability, low treatment adherence (2), and may become irritable and uncooperative when required to complete time-consuming measurement tools, impacting the accuracy of the results.

The PID Brief Form [PID-5-BF, 25 items, (9)] and the Short Form [PID-SF, 100 items, (27)] addressed this issue effectively by reducing the number of items. So far, the PID-5-BF has been translated and adapted into Brazilian Portuguese (28), American (29), French (30), Portuguese (31), Italian (32) and Chinese (33) versions, and proved to be reliable and valid in general. However, the criterion-related validity of its Antagonism factor for typical personality disorder types was not satisfactory in the sole Asian sample (33), which therefore needs further validation. Unlike the PID-5-BF, which targets the five higher-order domains (9), the PID-5-SF efficiently assesses both DSM-5-AMPD traits and facets (27). However, its psychometric properties have mainly been reported in European countries [(31, 34–37)], and its structure has been inconsistent across some of these reports (34–36). Hence, a self-report instrument for rapid pathological personality trait screening in Asian cultures is demanding to complement the PID-5 in clinic settings, especially when an informant is unavailable.

The Parker Personality Measure [PERM, (38)] is a reliable and validated instrument for assessing pathological personality in Western culture, containing 92 clinical descriptions of impaired personality functioning across 11 personality disorder types, ten of which are consistent with DSM-5 classifications (9). The mean Cronbach’s α values for its 11 factors were 0.81 and 0.83 by self-report and corroborative witness, respectively (38). Studies involving college students and clinical populations have further demonstrated the reliability and validity of its Chinese version (39), reporting good internal consistency (alpha coefficients > 0.70) and adequate criterion-related validity with other personality measures like the Five-Factor Normal Personality Questionnaire and the Zuckerman–Kuhlman–Aluja Personality Questionnaire (39–41). Interestingly, the PERM also reflects a five-factor dimensional structure of pathognomonic personality. The authors named these domains “entitled/dissocial,” “inhibited,” “borderline,” “schizoid/schizotypal” and “obsessional” (38). The first four essentially correspond to most of the personality disorder dimensions in the ICD-11 and the Borderline pattern specifier (2, 24) and the last partially reflecting a feature of the Antagonism domain in the DSM-5-AMPD (24). Thus, the PERM could facilitate an understanding of the transition from categorical to dimensional diagnostic systems and pathological personality characteristics in specific populations. However, no study has yet fully validated the measurement properties of the five-factor dimensional structure of the PERM when categorical diagnostic criteria were in widespread use.

In recent years, mental health issues such as anxiety, depression, and insomnia have been frequently reported among college students [e.g., (42, 43)], along with phenomena like suicide and self-injury [e.g., (44)], cyberbullying (45), and cyber-addiction (46). These issues indicate that some students experience persistent psychological symptoms leading to impaired interpersonal and social functioning, often associated with dysfunctional personality [e.g., (47)]. Furthermore, typical personality disorders account for a certain percentage of Chinese college students (48, 49), especially among those with internet addiction (50). Therefore, the college student population may well reflect the continuum of personality functioning impairment from normal personality to personality disorder. Considering their ability to understand the questionnaires and sample accessibility, we chose college students as subjects.

The aim of this study is to further refine the items with the highest factor loadings, develop the Short Form of PERM (PERM-SF), and validate its measurement properties in Chinese. This facilitates rapid assessment and early intervention for pathological personality traits, thereby reducing the economic and social burden on patients and families, and further optimizing the allocation of public health resources. We will also discuss whether its structure complies with the dimensional model of ICD-11 or DSM-5-AMPD, and cross-cultural stability of its factor connotations.

## 2 Methods

### 2.1 Participants

Sample 1 (*N* = 1,768; 937 women and 831men, aged 20.59 years ± 1.95, ranging from 16 to 27 years) and Sample 2 (*N* = 1,614; 887 women and 727 men, aged 20.38 years ± 1.59, 17 to 27 years) were recruited from one comprehensive university in Zhejiang Province, China in September 2021 to January 2023 and March 2023 to January 2024 with random sampling, respectively. Both samples completed the online electronic version of the questionnaire via Chinese public questionnaire platform “Questionnaire Star”.^1^ The exclusion criteria for the fillers were as follows: (1) incomplete completion of the questionnaire; (2) identical answers to all items; (3) less than 5 min to complete the questionnaire; or (4) a standardized score of more than 65 on the Lie factor of the PERM, indicating possible masking of psychological symptoms. After excluding these cases one by one (4.21, 1.55, 3.06, and 2.74%, respectively), the overall valid response rate to the questionnaire was 88.44%.

Additionally, a cohort of 339 subjects in Sample 2 was drawn from March to June 2023 to complete the Symptom Checklist-90 [SCL-90, (51)] to further assess the divergent validity of the PERM-SF. Given the convenience and accessibility of the study, 202 subjects from Sample 2 were randomly invited to retake the PERM-SF questionnaire, with a mean measurement interval of 64.40 (±21.05) days, to assess longitudinal measurement invariance. The purpose, voluntariness, and privacy measures of the study were clearly explained in the study guidelines (survey results would be used for research purposes only; names could be replaced by initials), and feedback on results based on the original PERM was provided according to the needs of the subjects.

### 2.2 Measurement instruments

#### 2.2.1 The Parker personality measure

The PERM (38) is a self-reported personality scale with 92 clinical symptom items and 10 validity items designed to assess 11 different functioning styles of personality disorder, including Paranoid (10 items), Schizoid (8 items), Schizotypal (5 items), Antisocial (10 items), Borderline (10 items), Performative (6 items), Narcissistic (8 items), Avoidant (10 items), Dependent (10 items), Obsessive-Compulsive (6 items) and Passive-Aggressive (9 items). Each item is measured on a 5-point Likert scale (1 = very unlike me, 2 = moderately unlike me, 3 = somewhat like and unlike me, 4 = moderately like me, and 5 = very like me). Higher PERM factor scores indicate more severe personality disorder functioning styles. In this study, we revised the Chinese version of PERM with the permission of Gordon Parker, the lead author of the original questionnaire. The PERM-SF was developed following the Chinese Version of the PERM (PERM-C) by Wang et al. (39), which has good reliability and validity in previous studies (39–41).

#### 2.2.2 The Symptom Checklist-90

The SCL-90 (51) is a self-rating scale consisting of 90 items assessing nine dimensions of symptoms, namely somatization, depression, paranoid ideation, anxiety, psychosis, anxiety, hostility, interpersonal sensitivity, and obsessive-compulsive symptoms. Each item is scored on a 5-point scale from 1 (no) to 5 (severe). A higher total score reflects greater overall severity of psychological distress in the past week. The scale has shown good psychometric properties in a population of Chinese college students (52), and its Cronbach’s α in this study was 0.982 (*n* = 339).

### 2.3 Statistical analysis

Structural validity, internal consistency, longitudinal measurement invariance, and construct validity of the PERM-SF were assessed in accordance with the COnsensus-based Standards for the selection of health status Measurement Instruments (COSMIN) guidelines (53–55). All analyses were conducted using SPSS 21.0, JASP 0.19.3 and R 4.5.1.

#### 2.3.1 Structural validity

Exploratory factor analysis (EFA) was performed on the 92 clinical symptom items of the PERM-C using Sample 1 to validate its factor structure. Data factorability was first assessed via the Kaiser-Meyer-Olkin (KMO) measure and Bartlett’s test of sphericity. Principal component analysis was subsequently conducted, with the optimal number of factors determined through combined application of eigenvalue criterion with scree plot inspection (56), and Horn’s parallel analysis (57). Varimax rotation (maximum variance method) accounted for potential factor correlations. Items were systematically excluded according to these criteria: (1) target loadings were less than 0.450; (2) cross-loadings were greater than 0.400 (58, 59); and (3) assignment to factors containing fewer than three items.

After excluding ineligible items, the remaining ones were evaluated to construct a revised structural model of the PERM-SF. Confirmatory factor analysis (CFA) was applied to validate the factor model in Sample 2 using the Diagonally Weighted Least Squares estimator in R4.5.1, with model fit assessed via goodness-of-fit indices and the root mean square error of approximation (RMSEA) (60, 61). If the comparative fit index (CFI) and the Tucker-Lewis index (TLI) were greater than 0.900, and the standardized root mean square residual (SRMR) and RMSEA were less than 0.080, the GOF metrics would be considered to be good, and the model would be considered good, and the model would be well-fitted (62). The finalized factor model would be used for further analyses.

#### 2.3.2 Internal consistency

Cronbach’s α and McDonald’s *ω* were used to assess internal consistency of the PERM-SF. However, since the use of α as an indicator of internal consistency suffered from several problems, e.g., inconsistency of results with hypotheses affecting the assessment (63), McDonald’s ω, which has less demanding assumptions and captured the effect of deleted items on overall reliability (64), was mainly considered in the current study.

#### 2.3.3 Longitudinal measurement invariance

The longitudinal measurement invariance (configural, metric, scalar, strict) of the PERM-SF was rigorously tested using longitudinal CFA with temporally repeated measurements to ensure scale stability over time. In configural invariance model, all parameters were free to test whether the proposed factor structure fitted uniformly across groups. In metric invariance model, the factor loadings of each group were constrained to be equal. In the scalar invariance model, the factor loadings and the observed variable intercepts were constrained to test whether they were equal across groups. Finally, in strict invariance model, factor loadings, intercepts, and residuals were constrained to be equal across groups. Longitudinal measurement invariance was assessed based on the variance of fit metrics such as CFI, TLI, and RMSEA (65). Models were considered acceptable if ΔCFI was less than 0.010, ΔTLI was less than 0.010, and ΔRMSEA was less than 0.015 (66–68).

#### 2.3.4 Test–retest reliability

Using data from 202 subjects in Sample 2, we assessed the test–retest reliability of the PERM-SF factors by calculating the Intraclass Correlation Coefficient (ICC) under a two-way random-effects model for absolute agreement. The level of agreement was interpreted according to Cicchetti’s (69) criteria: below 0.40 indicated poor agreement; 0.40–0.59, fair; 0.60–0.74, good; and 0.75–1.00, excellent. Per COSMIN guideline (55), reliability (ICC) > 0.7, Rating +.

#### 2.3.5 Construct validity

To assess divergent validity, bivariate Spearman correlations were examined. Specifically, correlations were calculated, respectively, between the PERM-SF factors (where a correlation of *r* > 0.50 was expected), and between these PERM-SF factors and the SCL-90 total score. |*r*| < 0.30 indicates a weak correlation, 0.30 ≤ |*r*| < 0.70 indicates a moderate correlation, and 0.70 ≤ |*r*| ≤ 1.00 indicates a strong correlation (70, 71). For factor intercorrelations, above 0.80 or 0.85 implies poor discriminant validity (72). Given the related yet dissimilar constructs of the PERM-SF and the SCL-90, the former assesses core personality traits and personality dysfunction, whereas the latter one screening for a broader range of psychological symptoms and their severity (52), a low to moderately significant positive correlation between the two was expected (73). On the other hand, the SCL-90-Revised Personality Severity Index (PSI), which is calculated with the total score of the subscales addressing interpersonal problems (i.e., interpersonal sensitivity, hostility, and paranoid ideation), indicating the severity of personality disorder (74). Inspired by studies related to the SCL-90-R PSI (75), a moderate positive correlation between the PSI index calculated using the SCL-90 and the PERM-SF was expected. Construct validity was deemed satisfactory if at least 75% of the correlations aligned with these expectations (55). Discriminant validity was further evaluated using the Heterotrait-Monotrait Ratio (HTMT), with values below 0.85 considered acceptable.

## 3 Results

### 3.1 Structural validity

Using the eigenvalue criterion and scree plot, EFA was conducted on Sample 1 (college students), initially yielding a six-factor structure with maximum rotation (KMO = 0.975; Bartlett’s test: χ^2^ = 79597.179, *p* < 0.001), which explained 44.73% of the total variance. Items 01, 05, 06, 07, 08, 09, 13, 16, 19, 21, 24, 25, 26, 31, 33, 34, 40, 41, 42, 43, 47, 48, 54, 61, 62, 64, 66, 68, 70, 71, 74, 76, 79, 81, 82, 87, 94, and 100 were subsequently excluded based on low factor loadings (<0.45) or substantial cross-loadings (>0.40). Analysis of the remaining 54 items revealed a stable five-factor structure (KMO = 0.963; Bartlett’s test: χ^2^ = 45010.892, *p* < 0.001), accounting for 49.06% of the total variance. As all retained items met the criteria for factor loading and cross-loading, the EFA supported a five-factor solution comprising 54 items. The factor composition was as follows: Factor I (F1): Items 14, 17, 18, 32, 38, 39, 46, 50, 51, 52, 53, 55, 56, 58, 59, 60, 63, 65, 67, 69, 77, 85, 95; Factor II (F2): Items 02, 03, 12, 15, 20, 22, 23, 36, 37, 45, 73, 78, 90, 91, 92, 99, 102; Factor III (F3): Items 72, 84, 86, 96, 97, 98; Factor IV (F4): Items 10, 83, 88, 93; Factor V (F5): Items 27, 29 (reverse-scored), 30, 49 (reverse-scored). Factor loadings are presented in Table 1. This refined instrument was designated the Short Form of PERM (PERM-SF; see Table 2 for item details). Parallel analysis initially suggested nine factors. However, applying the retention criteria (≥3 items with loadings > 0.45 and cross-loadings < 0.40) also supported a five-factor solution.

**Table 1:** Factor loadings for each item in the exploratory factor analysis (with Sample 1, *N* = 1,768).

Items (PERM factor)	Factor 1	Factor 2	Factor 3	Factor 4	Factor 5	Communalities
PERM 60 (Narcissistic)	**0.728**	0.153	0.105	0.053	0.019	0.568
PERM 58 (Narcissistic)	**0.689**	0.119	0.051	0.045	0.112	0.506
PERM 69 (Antisocial)	**0.685**	0.211	0.184	−0.043	0.004	0.550
PERM 50 (Antisocial)	**0.661**	−0.003	0.117	0.174	0.034	0.482
PERM 65 (Antisocial)	**0.622**	0.297	0.168	−0.163	0.049	0.532
PERM 56 (Narcissistic)	**0.596**	0.244	0.034	0.130	0.184	0.467
PERM 52 (Paranoid)	**0.590**	0.293	0.123	0.099	0.190	0.496
PERM 55 (Narcissistic)	**0.589**	0.265	0.084	0.051	0.265	0.497
PERM 67 (Paranoid)	**0.583**	0.331	0.162	0.072	0.164	0.508
PERM 59 (Histrionic)	**0.581**	0.175	0.099	0.040	0.306	0.473
PERM 51 (Paranoid)	**0.578**	0.311	0.130	0.186	0.091	0.490
PERM 77 (Narcissistic)	**0.567**	0.283	0.191	−0.049	0.105	0.452
PERM 18 (Passive-aggressive)	**0.557**	0.264	0.065	0.026	0.136	0.403
PERM 95 (Antisocial)	**0.556**	0.169	0.256	0.097	−0.048	0.415
PERM 85 (Narcissistic)	**0.555**	0.102	0.131	0.216	0.017	0.382
PERM 14 (Antisocial)	**0.551**	0.005	0.083	−0.109	−0.015	0.323
PERM 63 (Narcissistic)	**0.547**	0.205	0.276	0.064	0.104	0.431
PERM 53 (Paranoid)	**0.544**	0.293	0.099	0.193	0.179	0.460
PERM 39 (Histrionic)	**0.542**	0.012	0.174	−0.096	0.044	0.336
PERM 17 (Passive-aggressive)	**0.516**	0.231	0.145	−0.075	0.161	0.373
PERM 46 (Narcissistic)	**0.509**	0.310	0.053	0.015	0.074	0.363
PERM 32 (Histrionic)	**0.494**	0.228	0.022	0.164	0.220	0.372
PERM 38 (Antisocial)	**0.469**	0.308	0.209	−0.282	0.082	0.444
PERM 92 (Dependent)	0.165	**0.657**	0.113	−0.002	0.051	0.474
PERM 91 (Avoidant)	0.254	**0.654**	0.205	0.035	0.101	0.546
PERM 102 (Schizoid)	0.129	**0.628**	0.227	−0.099	−0.029	0.473
PERM 23 (Avoidant)	0.158	**0.624**	0.001	0.152	0.190	0.474
PERM 37 (Avoidant)	0.133	**0.613**	0.045	0.008	0.051	0.399
PERM 45 (Dependent)	0.218	**0.595**	0.033	−0.138	0.075	0.428
PERM 36 (Dependent)	0.125	**0.595**	0.046	−0.002	0.090	0.380
PERM 90 (Dependent)	0.179	**0.573**	0.013	0.174	−0.024	0.392
PERM 22 (Avoidant)	0.187	**0.558**	0.057	0.158	0.234	0.429
PERM 73 (Avoidant)	0.329	**0.554**	0.180	0.076	0.015	0.454
PERM 3 (Borderline)	0.089	**0.552**	0.019	−0.027	0.34	0.429
PERM 99 (Schizotypal)	0.187	**0.531**	0.293	0.030	0.074	0.409
PERM 12 (Dependent)	0.132	**0.523**	0.083	−0.102	0.161	0.334
PERM 15 (Avoidant)	0.229	**0.519**	0.10	0.136	0.116	0.364
PERM 78 (Schizoid)	0.237	**0.502**	0.30	0.048	0.158	0.426
PERM 20 (Borderline)	0.207	**0.498**	0.222	0.012	0.283	0.421
PERM 2 (Avoidant)	0.150	**0.496**	0.106	0.069	0.109	0.296
PERM 96 (Borderline)	0.169	0.098	**0.825**	0.009	0.096	0.728
PERM 84 (Borderline)	0.204	0.055	**0.781**	0.039	0.104	0.667
PERM 98 (Antisocial)	0.239	0.172	**0.753**	−0.028	0.025	0.655
PERM 97 (Schizoid)	0.184	0.279	**0.706**	−0.014	0.099	0.619
PERM 72 (Borderline)	0.266	0.297	**0.67**	0.083	0.177	0.646
PERM 86 (Schizotypal)	0.323	0.195	**0.602**	0.094	0.093	0.522
PERM 88 (Obsessive-compulsive)	0.029	0.110	0.031	**0.835**	0.010	0.712
PERM 93 (Obsessive-compulsive)	0.037	0.069	0.049	**0.828**	0.010	0.694
PERM 83 (Obsessive-compulsive)	0.12	0.032	0.081	**0.789**	−0.002	0.644
PERM 10 (Obsessive-compulsive)	0.087	−0.008	−0.034	**0.740**	−0.01	0.557
PERM 29 (Schizoid, reversely scored)	0.162	0.200	0.123	−0.026	**0.777**	0.686
PERM 30 (Borderline)	0.269	0.195	0.194	0.045	**0.747**	0.708
PERM 27 (Borderline)	0.234	0.282	0.167	0.061	**0.724**	0.690
PERM 49 (Schizoid, reversely scored)	0.118	0.191	0.036	−0.067	**0.677**	0.515
SS loadings	8.941	7.243	4.141	3.102	3.064	N/A
Proportion variance	0.166	0.134	0.077	0.057	0.057	N/A
Cumulative variance	0.166	0.300	0.376	0.434	0.491	N/A
Proportion explained	0.338	0.273	0.156	0.117	0.116	N/A
Cumulative proportion	0.338	0.611	0.767	0.884	1.000	N/A

Bold font indicates items loadings higher than 0.450; SS loadings, sum of the squared loadings; N/A, not applicable.

**Table 2:** Detailed items of the Short Form of Parker Personality Measure.

PERM item	Item content	Original factor
Factor 1: Dissociality		
14	Lying coming pretty easy to me	Antisocial
17	I tend to obstruct the efforts of others by failing to do my own share of the work	Passive-aggressive
18	Without any justification, I have a tendency to criticize people who are in positions of power	Passive-aggressive
32	I am rather demanding and vain at times	Histrionic
38	I am rather irresponsible	Antisocial
39	I can be quite flirtatious and self-dramatic	Histrionic
46	I tend to expect favors without feeling obliged to return them	Narcissistic
50	I tend to be somewhat manipulative	Antisocial
51	I tend not to trust other people’s motives	Paranoid
52	I tend to magnify minor problems with other people to be convinced that they are being malicious or treacherous	Paranoid
53	I tend to be mistrustful and be skeptical of the motives of others	Paranoid
55	I tend to envy others or believe that others are envious of me	Narcissistic
56	I tend to inflate my sense of importance	Narcissistic
58	I tend to exaggerate my own achievements and talents	Narcissistic
59	My emotions tend to be exaggerated	Histrionic
60	I tend to take advantage of others to achieve my own needs	Narcissistic
63	I tend to believe that I am special and should only associate with other special people	Narcissistic
65	I tend to disregard the truth by telling lies	Antisocial
67	I tend to question the loyalty of others without justification	Paranoid
69	I can bend the truth if I think it will benefit myself	Antisocial
77	I am rather egotistical and inconsiderable of others	Narcissistic
85	I have a sense of entitlement (i.e., expecting favorite treatment and that others will comply with my expectations)	Narcissistic
95	At times, I can be somewhat deceitful	Antisocial
Factor 2: Self-doubt and detachment		
2	I am unwilling to be involved with others unless I am certain of being liked	Avoidant
3	I have erratic and contradictory ways of coping with stress	Borderline
12	I have difficulty in making everyday decisions without a lot of advice and reassurance from others	Dependent
15	Although I desire close intimate relationships, I avoid them for fear of being foolish and ridiculed or exposed and shamed	Avoidant
20	I have rather persisting feelings of emptiness	Borderline
22	I often worry about things said or done	Avoidant
23	I worry about embarrassing myself in front of others	Avoidant
36	I base most decisions on what others think	Dependent
37	I am very reluctant to take personal risks or engage in new activities because that may prove to be embarrassing	Avoidant
45	I prefer to be told what to do rather than make choices	Dependent
73	I try to avoid any stress that may risk me feeling rejected or humiliated	Avoidant
78	I am very much of a loner	Schizoid
90	I have difficulty disagreeing with others for fear that I will get angry or that I will lose their support	Dependent
91	I avoid activities at work or social contact with others for fear of criticism, disapproval or rejection	Avoidant
92	I have difficulty initiating projects or doing things on my own due to a lack of self-confidence in my judgment and abilities	Dependent
99	I am extremely uncomfortable in social situations (a feeling that does not ease with knowing people), feeling somewhat fearful of others	Schizotypal
102	I have a general lack of vitality, being sluggish, nonspontaneous, and not very expressive	Schizoid
Factor 3: Disinhibition-negative affectivity		
72	I make rather desperate attempts to deal with feelings (real or imagined) that others have or may abandon me	Borderline
84	I have somewhat unusual way of speaking (e.g., being vague, somewhat beside the point, or excessively elaborate)	Borderline
86	I have some behaviors that are rather odd, eccentric or peculiar	Schizotypal
96	At times I can be suicidally impulsive, either in making gestures or engaging in self-mutilating behaviors	Borderline
97	Few, if any activities, provide me with pleasure	Schizoid
98	I tend to be irresponsible in meeting the needs of my spouse, children, or other family members	Antisocial
Factor 4: Anankastia		
10	I tend to work ahead of family and friends	Obsessive-compulsive
83	I am excessively devoted to work and productivity, somewhat to the exclusion of leisure and mixing with friends	Obsessive-compulsive
88	I do not consider a task finished until it is perfect	Obsessive-compulsive
93	I am a perfectionist to the extent that it interferes with completing the actual task	Obsessive-compulsive
Factor 5: Borderline pattern		
27	My mood is very unstable, with marked shift from normality to depression to overexcitement	Borderline
29	I am flat in my emotions (reversely scored)	Schizoid
30	I have very intense and changeable moods, ranging from being overly happy to depressed to irritable to anxious	Borderline
49	I tend to be emotionally detached (reversely scored)	Schizoid

**Figure 1:**
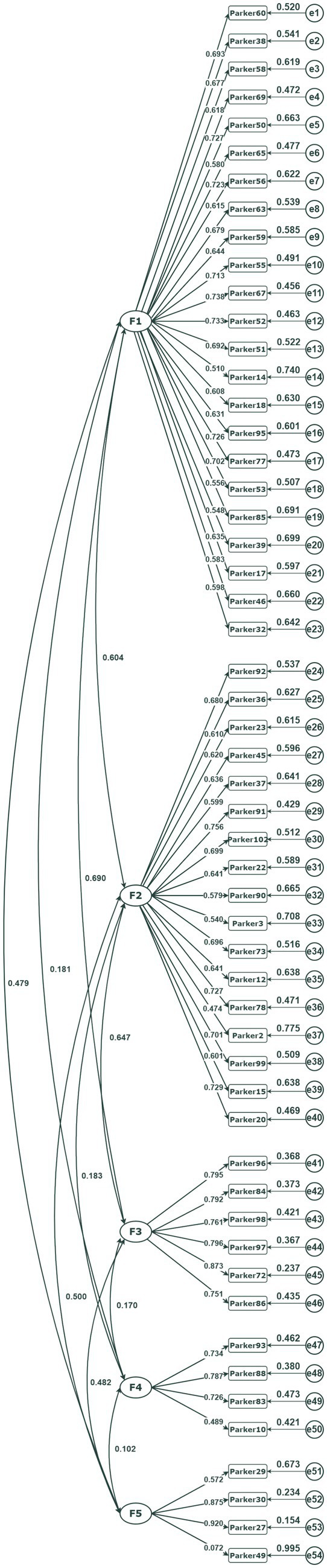
Standardized coefficients of confirmatory factor analysis results for a five-factor model of the Short Form of Parker Personality Measure (with Sample 2, *N* = 1,614).

CFA results for Sample 2 (college students) are presented in Table 3 and Figure 1. The five-factor PERM-SF model demonstrated a superior fit to Parker and Hadzi-Pavlovic’s (38) original 11-factor model, as evidenced by the following indices: CFI = 0.971, TLI = 0.970, SRMR = 0.059, and RMSEA = 0.063. In contrast, although formally identifiable, the 11-factor model suffered from severe statistical limitations, most notably near-perfect correlations between multiple latent variables (e.g., Histrionic–Narcissistic, *r* = 0.974). These high correlations indicate a lack of discriminant validity and directly contradict the model’s assumption of independent constructs. Given these issues, the more parsimonious five-factor model is statistically preferable. The five factors were named sequentially as Dissociality, Self-doubt-Detachment, Disinhibition-Negative Affectivity, Anankastia, and Borderline Pattern.

**Table 3:** Confirmatory factor analysis of alternative factorial solutions of the Short Form of Parker Personality Measure (with Sample 2, *N* = 1,614).

Model	CMIN	*df*	CMIN/*df*	CFI	TLI	SRMR	RMSEA (90% CI)
Five-factor (54 items)	10121.793	1,367	7.404	0.971	0.970	0.059	0.063 (0.062, 0.064)
11-factor (original 92 items)	37946.779	4,039	9.395	0.953	0.951	0.067	0.072 (0.072, 0.073)
Threshold	N/A	N/A	<3.000	≥0.900	≥0.900	<0.080	<0.080

CFI, comparative fit index; TLI, Tucker-Lewis index; SRMR, standardized root mean square residual; RMSEA, root mean square error of approximation; CI, confidence interval.

### 3.2 Internal consistency

Internal consistency estimates for the PERM-SF are detailed in Table 4. Both McDonald’s *ω* and Cronbach’s α demonstrated good internal consistency in Sample 1, with ω ranging from 0.834 to 0.932 and α from 0.832 to 0.932.

**Table 4:** Internal consistency of the Short Form of Parker Personality Measure (PERM-SF, with Sample 1, *N* = 1,768).

Variable	McDonald’sω (95%CI)	Cronbach’s α (95%CI)
F1 Dissociality	0.932 (0.927, 0.937)	0.932 (0.927, 0.937)
F2 Self-doubt-detachment	0.900 (0.893, 0.907)	0.901 (0.893, 0.907)
F3 Disinhibition-negative affectivity	0.881 (0.862, 0.896)	0.881 (0.853, 0.897)
F4 Anankastia	0.854 (0.843, 0.865)	0.852 (0.840, 0.863)
F5 Borderline pattern	0.834 (0.820, 0.847)	0.832 (0.819, 0.845)

This table shows ordinal forms of McDonald’s ω and Cronbach’s α.

### 3.3 Longitudinal measurement invariance

As presented in Table 5, measurement invariance testing in the Sample 2 subsample (*n* = 202) demonstrated all incremental fit index differences (ΔCFI, ΔTLI, ΔRMSEA) fell within established thresholds. These results suggest temporal stability of the measurement model across assessment points.

**Table 5:** Test of measurement invariance of the Short Form of Parker Personality Measure across time (with part of Sample 2, *n* = 202).

Model	CFI	ΔCFI	TLI	ΔTLI	RMSEA (90% CI)	ΔRMSEA
Configural	0.965		0.963		0.074 (0.071, 0.076)	
Metric	0.960	−0.005	0.959	−0.004	0.078 (0.075, 0.080)	0.004
Scalar	0.965	0.005	0.965	0.006	0.071 (0.069, 0.074)	−0.007
Strict	0.965	<0.001	0.965	<0.001	0.071 (0.069, 0.074)	<0.001
Threshold	≥0.900	<0.010	≥0.900	<0.010	<0.080	<0.015

CFI, comparative fit index; TLI, Tucker-Lewis index; RMSEA, root mean square error of approximation; CI, confidence interval; Δ, a change in CFI, TLI, and RMSEA.

### 3.4 Test–retest reliability

As shown in Table 6, two-way random-effects ICCs ranged from 0.488 to 0.759 for the five factors of PERM-SF, indicating acceptable test–retest reliability.

**Table 6:** Test–test reliability of the PERM-SF factors (with part of Sample 2, *n* = 202).

Variable	ICC value	95% CI	*F*	*p*
F1 Dissociality	0.747	0.679, 0.802	6.901	<0.001
F2 Self-doubt-detachment	0.759	0.694, 0.812	7.300	<0.001
F3 Disinhibition-negative affectivity	0.636	0.547, 0.712	4.501	<0.001
F4 Anankastia	0.488	0.376, 0.587	2.909	<0.001
F5 Borderline pattern	0.524	0.416, 0.617	3.203	<0.001

ICC, Intraclass Correlation Coefficient; CI, confidence interval.

### 3.5 Construct validity

As shown in Table 7, weak to moderate positive intercorrelations were observed among PERM-SF factors (rs = 0.104–0.630, Ps < 0.01), except for the non-significant association between Anankastia and Borderline Pattern (*r* = 0.047, *p* = 0.060). Additionally, each PERM-SF factor showed weak to moderate positive correlations with the SCL-90 total score and PSI (rs = 0.182–0.494, Ps < 0.01). Finally, discriminant validity was supported by the HTMT analysis, in which all values remained below the 0.85 threshold, as confirmed by bootstrap confidence intervals.

**Table 7:** Bivariate correlations and Heterotrait-Monotrait Ratio (HTMT) for the Short Form of Parker Personality Measure (PERM-SF) and Symptom Checklist-90 (SCL-90) (with Sample 2, *N* = 1,614; for correlations and HTMTs of SCL-90, with part of Sample 2, *n* = 339).

Variable		F1	F2	F3	F4	F5
F1 Dissociality	*r*	1				
F2 Self-doubt-Detachment	*r*	0.540**	1			
	HTMT (95%CI)	0.206 (0.187, 0.229)	–			
F3 Disinhibition-negative affectivity	*r*	0.630**	0.576**	1		
	HTMT (95%CI)	0.279 (0.251, 0.308)	0.250 (0.221, 0.278)	–		
F4 Anankastia	*r*	0.131**	0.133**	0.104**	1	
	HTMT (95%CI)	0.070 (0.045, 0.095)	0.068 (0.041, 0.096)	0.074 (0.042, 0.105)	–	
F5 Borderline pattern	*r*	0.357**	0.385**	0.368**	0.047	1
	HTMT (95%CI)	0.153 (0.130, 0.177)	0.164 (0.138, 0.190)	0.185 (0.156, 0.213)	0.029 (−0.004, 0.058)	–
SCL-90 total score	*r*	0.372**	0.494**	0.386**	0.182**	0.298**
	HTMT (95%CI)	0.147 (0.102, 0.203)	0.166 (0.120, 0.219)	0.231 (0.172, 0.306)	0.070 (0.021, 0.128)	0.112 (0.066, 0.159)
SCL-90 PSI	*r*	0.401**	0.451**	0.383**	0.197**	0.296**

**p < 0.01. PSI, Personality Severity Index; CI, Confidence Interval.

## 4 Discussion

This study investigated the measurement properties of the PERM-SF in a Chinese college student sample. Compared to the original PERM’s retention threshold [items with loadings ≥ 0.30; (38)], we implemented more stringent criteria, resulting in substantial item reduction. Notably, five-factor structure of the PERM-SF basically covered all the personality disorder dimensional features of ICD-11, including the borderline pattern specifier. This differs somewhat from the structure of the widely used PID (18), which captured different facets in both DSM-5-AMPD and ICD-11 criteria (76). Furthermore, the PERM-SF demonstrated acceptable internal consistency and construct validity while satisfying longitudinal measurement invariance requirements. Collectively, these findings provide preliminary evidence for the PERM-SF’s reliability and utility in assessing pathological personality dimensions.

### 4.1 Structural validity

EFA provided initial evidence for the PERM-SF’s five-factor structure, subsequently supported by CFA. The current factor composition demonstrated both continuity and expansion relative to Parker and Hadzi-Pavlovic’s (38) original PERM: F1 Dissociality retained narcissistic, antisocial, and histrionic features from the original dissocial factor, while incorporating additional passive-aggressive and paranoid traits. F2 Self-doubt-Detachment preserved dependent and avoidant characteristics akin to the original inhibited factor, but expanded to include schizoid, schizotypal, and borderline features. F3 Disinhibition-Negative Affectivity incorporated schizoid/schizotypal traits from the original counterpart, while integrating additional borderline and antisocial expressions. F4 Anankastia maintained obsessive-compulsive features consistent with the original factor. F5 Borderline Pattern conserved core borderline characteristics while assimilating schizotypal elements. This empirical alignment supports the structural plausibility of the five-factor personality disorder model. The observed trait overlaps across factors are consistent with diagnostic convergence between DSM-5-AMPD and ICD-11 dimensional frameworks (76). Notably, these findings echo Parker and Hadzi-Pavlovic’s (38) original observation that paranoid traits predominantly loaded onto Cluster B personality disorders.

The five factors in this study were under the dimensions of PD covered by the ICD-11, except that Factor 2 was changed from Interpersonal Detachment to Self-doubt-Detachment. Compared with that in the ICD-11, the PERM-SF Self-doubt-Detachment reflects social withdrawal, avoidance of intimacy, lack of self-confidence and reduced emotional expression but not indifference to others. This suggests that in Chinese culture, self-doubt or low self-confidence rather than emotional indifference is the primary cause of interpersonal detachment in individuals with pathological personality traits. This may lead to uncertainty about the attitudes of others in interpersonal relationships and fear of being devalued, resulting in a tendency to distance oneself from others. Cross-cultural studies of social behavior have also found that people with self-doubt who were different from the dominant culture tended to be more reserved and averse to stares (77), and that the expression of social anxiety in the context of cultural difference was related to the degree to which one defined him/herself as independent or in need of interdependence (78). This finding also made a clearer distinction between Factor II and Factor I (Dissociality) in the PERM-SF compared to the two factors Dissociality and Detachment in the ICD-11, i.e., they reflect more passive and more active causes of interpersonal problems, respectively. In addition, the Disinhibition and Negative Affectivity factors were combined into Factor III, which embodied impulsivity without regard to risks and consequences, irresponsibility, and negative emotional feelings such as loss of pleasure and feelings of abandonment, but not distraction. While there was already a precedent for combining Impulsive and Borderline into Emotionally unstable in ICD-10 (2), the results of the present study suggested a stronger association between lack of inhibition and negative affectivity rather than the borderline pattern, which contributed to pathological personality in Chinese culture. Previous evidence also suggested a strong relationship between the Inhibition domain and its various aspects and negative affect such as anxiety and depression (79, 80). Factor IV Anankastia in the current study exemplifies behavioral control over self, emotional discipline and perfectionism, but not control over others. Previous research has found a bipolar relationship between Anankastia and Disinhibition (81), and the emphasis on social role obligations and the need for hierarchical obedience relationships in traditional Chinese Confucian culture may make socially maladjusted individuals more prone to internal emotion regulation in order to conform to social norms (82–84). The borderline pattern was the fifth factor that embodied unstable emotions, but not unstable interpersonal relationships and self-image. This was partly because the latter was more highly correlated with Factor 2, and partly because Chinese culture was subtle and implicit and promoted a high degree of interpersonal harmony (85), which made individuals more inclined to minimize the external expression of their emotions to avoid possible damage to their relationships (86).

Notably, prior research extracting metrics from three personality instruments (PERM-C, Five-Factor Nonverbal Personality Questionnaire, Zuckerman-Kuhlman Personality Questionnaire) in Chinese populations similarly identified a five-factor structure (39). The current solution demonstrates both convergence and divergence with this earlier model. Namely, Dissocial (FI), Inhibition (FIV), and Compulsivity (FV) align with pathological dimensions identified here; Emotional Dysregulation (FIII) is superseded by the Borderline Pattern; Experience Seeking (FII), which showed minimal PERM-C loadings previously, is replaced by Self-doubt-Detachment. These structural differences may stem from measurement focus variation. Whereas the items of Wang et al. (39) primarily captured normal personality variation, the current instrument exclusively operationalizes pathological personality descriptors.

### 4.2 Internal consistency

The PERM-SF demonstrated good internal consistency, with both McDonald’s *ω* and Cronbach’s α coefficients exceeding 0.80 for all five factors, indicating acceptable reliability. Importantly, item reduction enhanced reliability coefficients compared to the original PERM. Thus, given its psychometric properties and assessment efficiency, the PERM-SF represents a psychometrically sound option when time constraints prioritize brief self-assessment protocols.

### 4.3 Longitudinal measurement invariance

Longitudinal measurement invariance of the PERM-SF was supported through the longitudinal CFA. Model comparisons revealed non-significant Δχ^2^ values across configural, metric, scalar, and strict invariance levels, while ΔCFI, ΔTLI, and ΔRMSEA remained within established thresholds (67). As preliminary evidence for temporal stability, the current measurement intervals (40 days to 3 months) provide a foundation for subsequent invariance studies, though future research should systematically vary retest durations. Further validation across broader age cohorts would strengthen invariance evidence.

### 4.4 Test–retest reliability

The temporal stability of the scale was assessed using a two-month test–retest interval in a subsample of 202 participants. The resulting ICCs indicate a moderate level of reliability. While these values are more modest than the excellent stability reported for established measures like the PID-5 over both short and extended periods (87), they fall within the acceptable range for the initial validation of a novel personality instrument. It may be attributable to our longer retest interval than the typical one-week interval used in many high-reliability benchmarks, which allows for greater natural fluctuation, and the potential state-sensitivity of the constructs measured.

### 4.5 Construct validity

The weak-to-moderate positive intercorrelations among PERM-SF factors, alongside similar associations between all factors and SCL-90 PSI/total scores, collectively indicate adequate discriminant validity and sensitivity to pathological personality severity, as evidenced through interpersonal dysfunction and psychological distress markers of SCL-90. No significant association was observed between the PERM-SF Borderline Pattern and Anankastia factors. Notably, the Borderline Pattern is characterized by core traits of high disinhibition and negative affectivity (24). First, Borderline Pattern and Anankastia map onto orthogonal dimensions within the big-five traits, specifically, high Neuroticism with low Conscientiousness versus high Conscientiousness (24, 81). This structural independence has been replicated in international samples (88). Second, within Chinese cultural contexts, this distinction may be further amplified by cultural norms that promote control-oriented (Anankastic) coping strategies while suppressing disinhibited emotional expression (associated with the Borderline Pattern) (82–84). In general, the PERM-SF demonstrates acceptable construct validity.

A key finding of this study is the established discriminant validity among the PERM-SF factors. Although some factors were moderately to highly correlated—reflecting shared variance within the personality pathology spectrum—the HTMT supported their statistical distinctiveness. For example, despite a substantive correlation between Dissociality and Disinhibition-Negative Affectivity, their distinction met modern psychometric standards for discriminant validity (89). These results indicate that the PERM-SF effectively captures multifaceted yet discrete aspects of personality pathology in Chinese college students.

### 4.6 Limitations

Several limitations warrant consideration when interpreting the findings of this study. First, while initial validation focused on college students, a population demonstrating non-negligible personality disorder prevalence (48, 49) and frequent personality-related functional impairment (47), generalizability requires verification across clinical samples, diverse age cohorts (e.g., adolescents, older adults), and varied cultural contexts. This necessity is underscored by documented cross-cultural variability in personality disorder manifestation (9). Second, to reduce participant burden, we did not administer the classical personality disorder measures that support the DSM-5-AMPD or the ICD-11 criteria, such as the PID-5 (18). This constraint limited comprehensive evaluation of convergent and discriminant validity. Third, although the present study allowed for anonymous completion and the PERM questionnaire had a separate Lie factor for screening to improve the validity of the results, the possibility of subject reporting bias could not be ruled out, as the questioning mainly involved negative descriptions of personality. Fourth, the test–retest reliability warrants further investigation with larger samples and varying retest durations. Future validation should therefore incorporate multi-method assessments, particularly observer-rated measures of personality pathology dimensions and severity in broader and more diverse samples.

## 5 Conclusion

In this study, we developed the PERM-SF and established its substantial alignment with the core dimensions of personality disorders outlined in the latest ICD-11. Its measurement properties, including structural validity, internal consistency, longitudinal measurement invariance, and construct validity, were successfully validated within a sample of Chinese college students. The significantly abbreviated item count of this short form renders it particularly well-suited for the efficient screening of pathological personality traits. Further validation, however, is warranted in clinical populations and broader community samples. The application of the PERM-SF holds considerable promise that it could facilitate the early detection and effective treatment of PD while simultaneously offering new avenues for research and intervention into common mental illnesses and serious psychosocial problems (e.g., suicide and self-harm), optimizing the distribution of finite public health resources.

## Data Availability

The original contributions presented in the study are included in the article/supplementary material, further inquiries can be directed to the corresponding authors.

## Footnotes

^1^ https://www.wjx.cn/

## Glossary

CFA: Confirmatory factor analysis
CFI: Comparative fit index
COSMIN: COnsensus-based Standards for the selection of health status Measurement Instruments
DSM-5: Diagnostic and statistical manual of mental disorders, fifth edition
DSM-5-AMPD: DSM-5 Section III (the alternative model)
EFA: Exploratory factor analysis
GOF: Goodness of fit
HTMT: Heterotrait-Monotrait ratio
ICC: Intraclass correlation coefficient
ICD-11: International classification of diseases-11
KMO: Kaiser-Meyer-Olkin
PD: Personality disorder
PERM: Parker personality measure
PERM-C: Chinese version of the Parker personality measure
PERM-SF: Short form of the Parker personality measure
PID-5: Personality inventory for DSM-5
PID-5-BF: Personality inventory for DSM-5-brief form
PID-5-SF: Personality inventory for DSM-5-short form
PSI: Personality severity index
RMSEA: Root mean square error of approximation
SCL-90: Symptom Checklist-90
SRMR: Standardized root mean square residual
TLI: Tucker-Lewis index
